# A common tumor in an uncommon site: epithelioid Leiomyoma arising from the seminal vesicle—a case report

**DOI:** 10.1186/s12894-022-00963-3

**Published:** 2022-01-29

**Authors:** Xiaoyang Xu, Shuang He, Yangyang Li, Feifei Wen, Lizhen Lu, Zhongze Cui, Shuhua Wu

**Affiliations:** Department of Pathology, Binzhou Medical University Hospital, Binzhou, 256603 Shandong Province China

**Keywords:** Seminal vesicle, Epithelioid leiomyoma, Case report, Pathology, Computed tomography

## Abstract

**Background:**

Leiomyoma of the seminal vesicle is a rare leiomyoma characterized by the formation of benign leiomyomatous tissue within the seminal vesicle. Although histologically benign, excessive size can lead to urinary system disease if left untreated. Herein, we report a case of a seminal vesicle epithelioid leiomyoma.

**Case presentation:**

A 36-year-old Chinese man sought medical attention at our hospital for urination pain and hemospermia. CT showed a 5.3 cm × 5.0 cm seminal vesicle mass with a mixed density in the right seminal vesicle. The gross specimen showed light yellow, gray, and white tissues, with softness and hemorrhage in some places. Histologically, it showed classic spindle cell proliferation, with spindle cells arranged in fascicles, and mitosis was rare. Immunohistochemistry showed frequent expression of smooth muscle markers, such as calponin, SMA, and desmin. A diagnosis of epithelioid leiomyoma was proposed according to the immunohistochemical findings and morphology. The patient did not receive adjuvant therapy. There was no evidence of tumor recurrence in the 10 months after surgery.

**Conclusions:**

We report the first case of epithelioid leiomyoma in the seminal vesicle. This disease should be included in the differential diagnostic list of seminal vesicle tumors with epithelioid morphology.

**Supplementary Information:**

The online version contains supplementary material available at 10.1186/s12894-022-00963-3.

## Background

Primary tumors of the seminal vesicle are rare. Seminal vesicle cysts occur more commonly than solid masses. Among solid masses, the most common primary tumor is adenocarcinoma. Among them, the most common is metastatic adenocarcinoma from colorectal or prostate. Benign solid tumors of the seminal vesicle include cystadenoma of the seminal vesicles, fibroadenoma, solitary fibrous tumor and leiomyoma. Among the seminal vesicle masses, leiomyoma is a very rare mass, with only a few cases reported in the literature. We searched PubMed using the keywords “leiomyoma”, “seminal vesicle”, “seminal duct”, “seminal vesicle pathology” and “seminal duct pathology”. As of January 2021, only 12 cases of seminiferous duct/scrotal leiomyoma have been reported in the medical literature, but no epithelioid leiomyoma has been reported. We report a new case of primary epithelioid leiomyoma of the seminal vesicle and present a brief review of the literature.

## Case presentation

### Clinical summary, radiological findings and gross examination

A 36-year-old Chinese man sought medical attention at our hospital for urination pain and hemospermia. The patient developed hematospermia 2 weeks prior. At the same time, there was pain in urination, and the pain was a tingling sensation. His symptoms were relieved after urination. There were mixed lesions behind the bladder by B-ultrasonic examination (no image provided). No special disease history was reported.

**Fig. 1:**
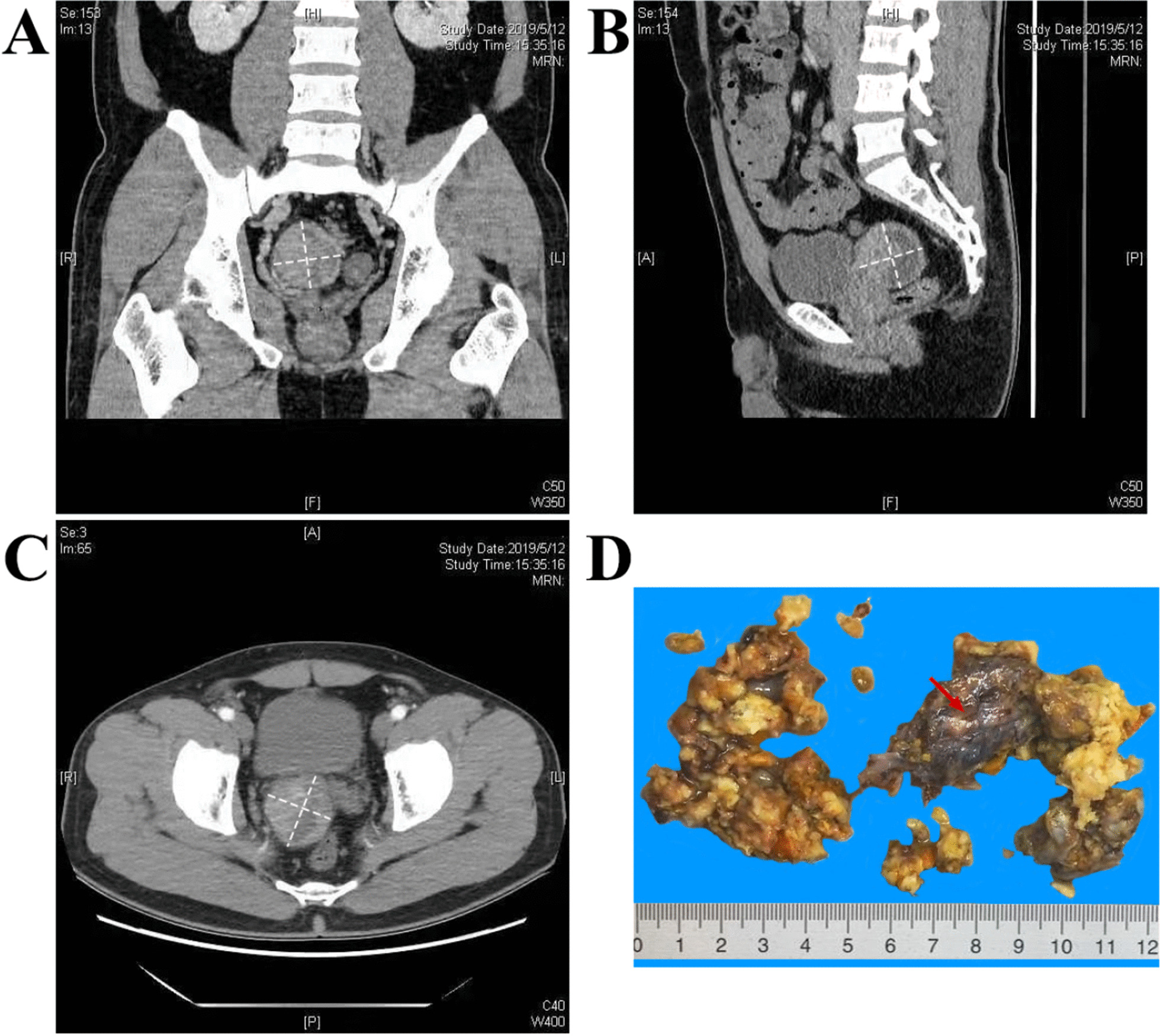
Clinical imaging findings. **a**–**c** CT examination revealed a mixed density mass with a clear boundary. **d** Macroscopically, the tumor was an 80 × 55 × 2-mm mass with a fibrous capsule (red arrows)

Computed tomography (CT) showed that the mixed density of mass could be seen in the right seminal vesicle with clear boundary. The size of the mass was 5.3 cm × 5.0 cm. CTU (CT urography) showed that the mass had uneven enhancement, and patchy non enhancement areas could be seen (Fig. 1a–c). Preoperative diagnostic imaging described a “space-occupying right seminal vesicle, potentially malignant disorder”. Next, the patient underwent laparoscopic excision of the seminal vesicle tumor.

Due to the nature of laparoscopic surgery, the specimen was relatively broken. However, it seemed that there was still a fibrous capsule in some places (Fig. 1d). In other word, the boundary of the mass in vivo was relatively clear. The gross specimen showed light yellow, gray, and white tissues, with solid and soft.

### Histological characteristics

**Fig. 2:**
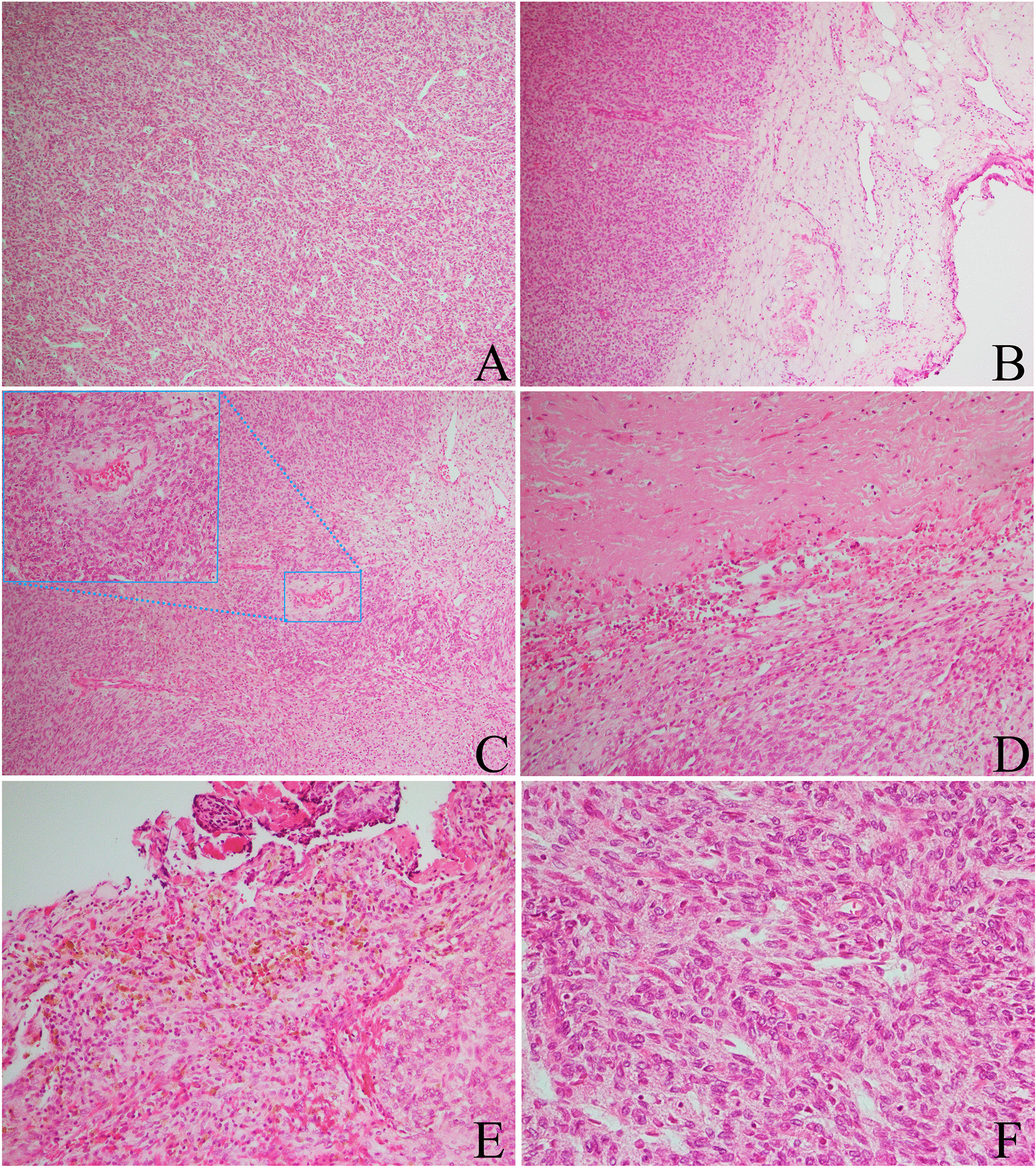
Histology of the seminal vesicle tumor (H&E staining, by OLYMPUS BX53). **a** The tumor cells are dense and arranged like strips (× 4). **b** The tumor has a fibrous capsule and the boundary is relatively clear (× 4). **c** Tumor cells line up around blood vessels to form a perivascular tumor-like structure (× 4). **d** The collagen with hyaline degeneration could be seen in stroma (× 10). **e** Old bleeding can be seen around the tumor (× 10). **f** The tumor is composed of round or polygonal cells and the cytoplasm is eosinophilic or transparent (× 20)

Microscopically, the mass was rich in cells, there were fibrous capsules, and the boundary was relatively clear. The cells were arranged in the shape of sheets, nests or strips. The tumor cells were round and partly polygonal, with an abundant and acidophilic cytoplasm. The nucleus was located in the center of the cell. Mitosis was rare (< 2/10 HPF). Some tumor cells lined up around blood vessels to form a perivascular tumor-like structure. Vascular hyperplasia was seen in the stroma (Fig. 2). We found hemosiderin deposits around the tumor, indicating that there was bleeding, which may be the reason for the patient’s hemospermia syndrome.

### Immunohistochemical findings

**Fig. 3:**
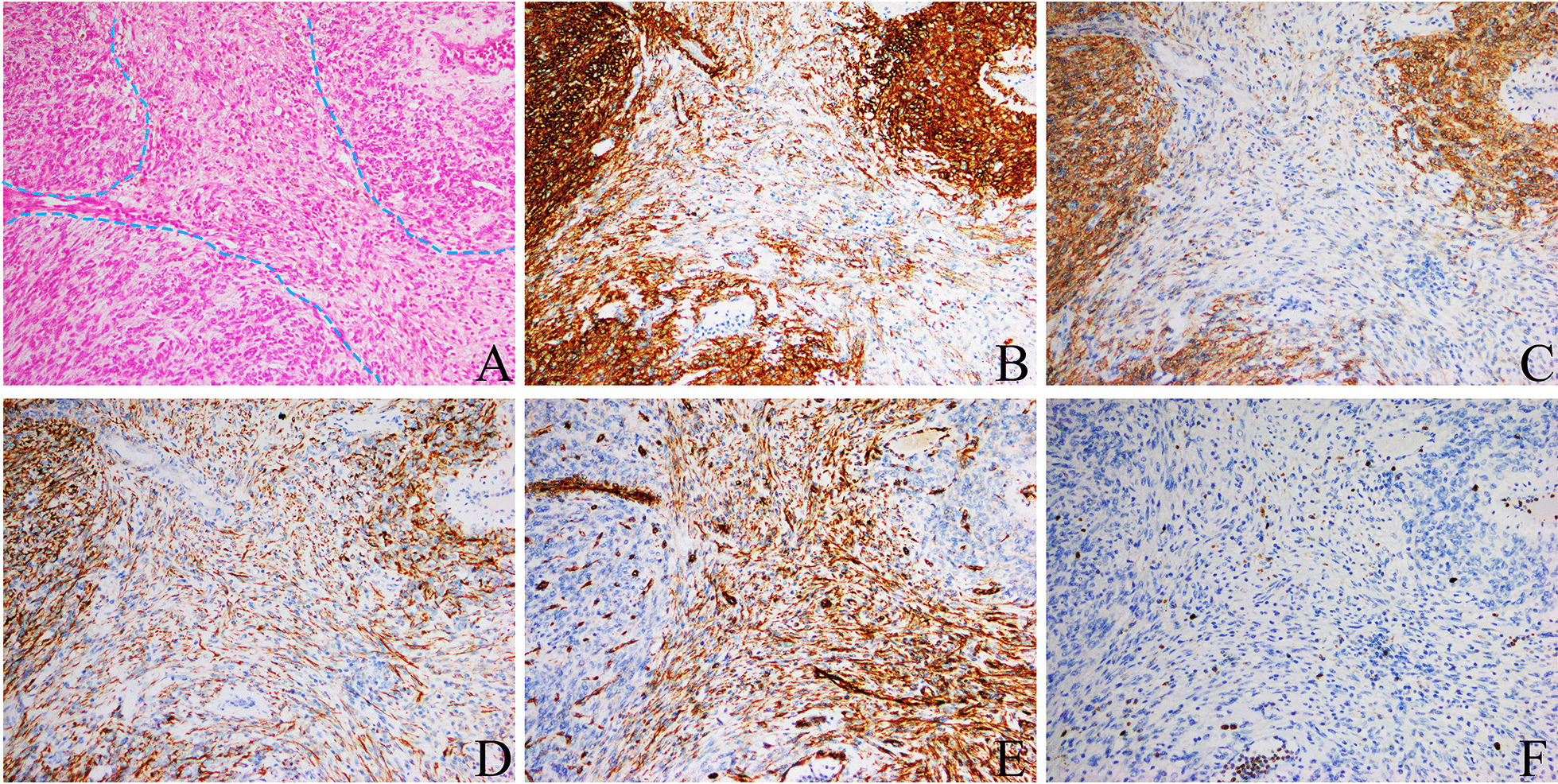
Immunohistochemistry of seminal vesicle tumor (Envision, × 10, by OLYMPUS BX53). **a** H&E staining showed that the tumor cells were denser than interstitial cells. IHC findings showed that most tumor cells were strongly positive for calponin (**b**) and moderately positive for SMA (**c**) and desmin (**d**). None of the tumor cells showed Vimentin expression, while nonneoplastic endothelial cells expressed Vimentin (**e**). The positive rate of Ki-67 (**f**) was low

The immunohistochemical findings were CK (locally positive), Vimentin(−), calponin(+), SMA(+), Desmin(+), CK5/6(−), P63(−), HMB45(−), MelanA(−), EMA(−), 34βE12(−), CD31(−), CD34(−), ERG(-), D2-40(−), S-100(−), CD117(−), DOG-1(−), CD68(−), PSA(−), and Ki-67 (2–5%) (Fig. 3 and Additional file 1: Fig. S1).

Interpretation of the IHC results. PSA and ERG were negative to rule out metastasis of poorly differentiated prostate cancer. We could exclude low differentiated squamous cell carcinoma because P63 and 34βE12 were negative. Vascular markers were negative (e.g., CD31, CD34, D2-40, and ERG); therefore, vascular tumors were excluded. HMB45(−), Melan-A(−) and S-100(−) can exclude malignant melanoma. It is not a neurogenic tumor because S-100 was not expressed. GISTs were excluded because CD117, DOG-1 and CD34 were all negative. Ewing’s sarcoma was not considered due to ERG negativity. The immunohistochemistry results for smooth muscle markers, such as calponin, SMA, and desmin, were positive. Therefore, the proposed diagnosis was that the patient had a smooth muscle tumor. EMA and CD68 were used to differentiate fibrous histiocytoma from epithelioid leiomyoma.

## Discussion and conclusions

Leiomyoma often occurs in the uterus [1]. It is the most common tumor in the uterus and predominantly originates from smooth muscle. Epithelioid leiomyoma is one of the distinct cell signature subtypes. Epithelioid leiomyoma is mainly composed of round or polygonal cells [2]. The cytoplasm is eosinophilic or transparent. There is a round nucleus and delicate chromatin. Occasionally, the nucleolus can be seen. The cells are arranged in bands, strips and nests. Sometimes tumor cells seem to adhere to each other and occasionally line up around blood vessels to form a perivascular tumor-like structure. Epithelioid cells are the main components. Collagen with hyaline degeneration could be seen in the stroma. Studies have found that the cytoplasmic transparency is due to sublethal damage to cells, such as the vacuolation of mitochondria and lysosomes. In some cases, clear cells may be arranged in small nests or tubules, and they could form plexiform tumorlets. Plexiform tumorigenesis is easily misinterpreted as metastatic cancer, particularly metastatic invasive lobular carcinoma (ILC). Most notably, there are cases of death caused by epithelioid leiomyoma in the uterus. Therefore, we believe that epithelioid leiomyoma should be classified as a smooth muscle tumor considering its unique clinicopathological characteristics and malignant potential.

Diseases of the seminal vesicle are extremely rare, and there are only a few reports in the literature. A systematic review of the medical literature shows that only 12 cases of leiomyoma of the seminal vesicle have been reported so far (Table 1). All the reports agree that it is a benign tumor, and there were no recurrences or metastases reported. The median age of the patients was 65 years, and the youngest age of onset was 36 years. When the tumor is small, there are no spontaneous symptoms. However, when the tumor is more than 10 cm, it can cause lower abdominal pain and urinary symptoms. For radiologic examination, CT or MRI investigation is necessary. In 12 cases found in literature review, 10 cases underwent a radiological clarification either with CT or with MRI had been performed before any further intervention. In 2 cases based on MRI, the definitive diagnosis such as a solitary fi brous tumor or leiomyoma could be suspected, but at the end in all these cases, the diagnosis was only confirmed after the extirpation. At present, there are no publications on systematic radiology standards that can be used for the examination of such cases.

**Table 1:** Reported cases of leiomyoma of the seminal vesicle

No.	Year	Author	State	Age	Symptoms	Size (cm)	Position	Preoperative diagnosis	Mode of operation	The imaging
1	1944	Plaut [3]	USA	66	Left lower abdominal tumor	14 × 11	No record	No record	Laparotomy through a lower midline incision	No record
2	1990	Bahn DK [4]	USA	61	Asymptomatic	2	No record	Transrectal biopsy leiomyoma	No record	Endorectal ultrasound showed that a well-defined hypoechogenic mass lesion can be seen in the seminal vesicle
3	1994	Gentile [5]	USA	66	Asymptomatic	5 × 5	Left	Transrectal biopsy benign bland collagenous fibrous tumor	Laparotomy through a lower midline incision	CT of pelvis showed ​soft tissue mass deforming left posterolateral aspect of bladder. MRI showed low signal intensity mass
4	1996	Ahmadzadeh [6]	Germany	69	Dysuria and pollakiuria	5.0 × 4.5	Left	Transrectal biopsy no malignant cells	Laparotomy through a lower midline incision	Ultrasonography reveals a mass in the area of the left seminal vesicle.A computerized tomography scan shows a unilateral enlargement of the seminal vesicle on the left side
5	2007	Lallemand [7]	Belgium	52	Bladder outlet obstruction	4 × 3	Right	No record	Laparoscopic excision	CT showed a mass lesion in the right retrovesical region. The MRI revealed low signal intensity, well-marginated, ovoid mass in the right retrovesical located in the right seminal vesicle
6	2009	Shiotani [8]	Japan	74	Left hemilumbago	5.5 × 4.4	Left	Transrectal biopsy benign bland collagenous fibrous tumor	Laparotomy through a lower midline incision	CT revealed a calcifi ed soft tissue-density mass with coarse, clear margins located adjacent to the urinary bladder and seminal vesicles. Magnetic resonance imaging (MRI) revealed a mass of low and isointensity signals compared with soft tissue on T1-weighted imaging and low, moderately high, and isointensity signals on T2-weighted imaging
7	2009	Inan [9]	Turkey	74	Asymptomatic	1.2	Left	Transrectal biopsy leiomyoma	No record	The lesion was homogeneously hypo-intense on T2-weighted images and isointense on T1-weighted images homogeneous. After I.V contrast injection, the lesion enhanced strongly and homogeneously
8	2013	Shaikh [10]	India	63	Lower urinary tract symptoms	5.7 × 5.1	Left	Transrectal biopsy leiomyoma	Laparoscopic excision	MRI showed the presence of a retroperitoneal and retrovesical solid mass
9	2014	Miyajima [11]	Japan	65	Lower abdominal disobedience	9.3 × 4.4	Right	Transrectal biopsy leiomyoma	Laparotomy through a lower midline incision	CT showed oval plump tumor without contrast effect. MRI showed that the tumor was depicted with the same low signal as muscle in T1 and T2 stressed images. It was suspected to be myogenic tumor and isolated fibrous tumor derived from myofibroblasts
10	2016	Arnold [12]	African American	55	Treatment for prostate cancer	1.5 × 1.5	Left	No record	Robot assisted laparoscopic prostatectomy	No record
11	2018	Oliveira [13]	Brazil	60	Asymptomatic	4.0	Right	Transrectal biopsy leiomyoma	No record	Transabdominal pelvic ultrasound showing a well-defined solid hypoechoic lesion in the right seminal vesicle space.T2-weighted MRI sequence showing a well-defined, heterogeneous expansile lesion with predominantly low signal intensity. T1-weighted fast spin-echo MRI sequence showing a solid heterogeneous lesion with its epicenter in the right seminal vesicle and a predominantly isointense signal
12	2019	Mendrek [14]	Germany	41	Strong lower abdominal pain with unusually sudden onset	7.5 × 6.5	Right	Transrectal biopsy showed tissue without signs of malignancy	Laparotomy through a lower midline incision	CT showed a solid mass, which in sagittal plane was localised between urinary bladder and rectum and had well-defined boundaries but vague origin
13	2021	Present study	China	36	Urination pain and hemospermia	5.3 × 5.0	Right	No record	Laparoscopic excision	CT showed inconsistent density of the mass, and CTU showed uneven enhancement, and patchy non enhancement areas

This is the first report of epithelioid leiomyoma in the seminal vesicle, and the origin of the mass was identified by several groups of immunohistochemical markers. The most common treatment for seminal vesicle leiomyoma is surgery, which can not only relieve symptoms but also provide a clear diagnosis. In previous reports, open surgery was most frequently performed, and a few patients underwent laparoscopic resection. To date, there have been no reports of recurrence of leiomyoma in the seminal vesicle. The author will continue to follow up this case.

## Supplementary Information

**Additional file 1: Fig. S4** The immunohistochemical findings were SMA(+), calponin(+), Ki-67 (2–5%), CK (locally positive), PSA(−), ERG(−), CK5/6(−), P63(−), 34βE12(−), HMB45(−), MelanA(−), S-100(−), CD68(−), EMA(−), CD117(−), DOG-1(−),CD34(−), D2-40(−) and CD31(−).

## Data Availability

All data generated or analyzed during this study are included in this published article and its supplementary information files.

## Abbreviations

CK: Cytokeratin
CT: Computed tomography
SMA: Smooth muscle actin
EMA: Epithelial membrane antigen
