# Supplementary material for: A common tumor in an uncommon site: epithelioid Leiomyoma arising from the seminal vesicle—a case report
Source: BMC Urol. 2022 Jan 29;22:9. doi: 10.1186/s12894-022-00963-3 (PMC8800249; doi:10.1186/s12894-022-00963-3)
Supplement: Supplementary file 1 — Additional file 1: Fig. S4 The immunohistochemical findings were SMA(+), calponin(+), Ki-67 (2–5%), CK (locally positive), PSA(−), ERG(−), CK5/6(−), P63(−), 34βE12(−), HMB45(−), MelanA(−), S-100(−), CD68(−), EMA(−), CD117(−), DOG-1(−),CD34(−), D2-40(−) and CD31(−). [file 12894_2022_963_MOESM1_ESM.doc]

**Additional file 1: Fig. S1.** The immunohistochemical findings were SMA(+), calponin(+), Ki-67 (2–5%), CK (locally positive), PSA(−), ERG(−), CK5/6(−), P63(−), 34βE12(−), HMB45(−), MelanA(−), S-100(−), CD68(−), EMA(−), CD117(−), DOG-1(−),CD34(−), D2-40(−) and CD31(−).
